# Role of neutrophil extracellular traps against soft tissue infections

**DOI:** 10.1186/cc14157

**Published:** 2015-03-16

**Authors:** N Yamamoto, M Ojima, S Hamaguchi, T Hirose, R Takegawa, N Matsumoto, T Irisawa, M Seki, O Tasaki, T Shimazu, K Tomono

**Affiliations:** 1 Division of Infection Control and Prevention, Osaka University Graduate School of Medicine, Suita, Japan; 2 Osaka University Graduate School of Medicine, Suita, Japan; 3 Nagasaki University Graduate School of Biomedical Sciences, Nagasaki, Japan

## Introduction

Neutrophils work as the frontline of defense against infections and neutrophil extracellular traps (NETs) are one of the immune systems to suppress dissemination of infection by the netted chromatin decorated with antibacterial molecules. It was reported that NETs play an important role in various kinds of infections such as pneumonia. However, there is no report on NETs in patients of soft tissue infections. In this study, we evaluated NET production in pus and clarified the role of NETs as a host defense in patients of soft tissue infections.

## Methods

This study was conducted in the ICU of the Trauma and Acute Critical Care Center at Osaka University Hospital. We collected pus from the patients of soft tissue infections at the time when drainage or debridement was performed and when clinical improvement was observed. The smears of pus specimens were examined by immunohistochemistry to visualize the major NET components: DNA, neutrophil elastase, and histone H1. Concurrently, the patients' clinical data and laboratory data of blood were recorded to analyze the relationship with NET production.

## Results

A total of five patients were included in this study and drainage of abscess or debridement of infection site was performed in all of the cases. Four patients of them were diagnosed as necrotizing soft tissue infections by *Clostridium *spp. (*n *= 1) and *Bacteroides *spp. (*n *= 3) and the other was diagnosed as cervical abscess by *Streptococcus *spp. In all cases, no NETs but neutrophils were identified in the first pus: however, NETs appeared in the later smears as the patients' condition was getting better.

## Conclusion

These results suggested that NETs also worked as an immune system against soft tissue infections. Drainage or debridement of infection focus might promote NET production.

